# Editorial: Immune Cell Migration in Health and Disease

**DOI:** 10.3389/fimmu.2022.897626

**Published:** 2022-04-13

**Authors:** Jörg Renkawitz, Emmanuel Donnadieu, Hélène D. Moreau

**Affiliations:** ^1^ Biomedical Center (BMC), Walter Brendel Center of Experimental Medicine, Institute of Cardiovascular Physiology and Pathophysiology, Klinikum der Universität, Ludwig-Maximilians University (LMU) Munich, Munich, Germany; ^2^ Université de Paris, Institut Cochin, INSERM, CNRS, Paris, France; ^3^ Institut Curie, PSL University, Inserm U932, Immunity and Cancer, Paris, France

**Keywords:** cell migration, cytoskeleton, adhesion, monocytes, macrophages, neutrophils, T cells, dendritic cells

Migration of immune cells is essential to virtually every step of their surveillance function (1–6). In this Research Topic, we gather reviews and original research articles that emphasize the wide variety of both physiological and pathological contexts in which immune cell migration is either favored or impeded and exemplify the latest mechanistic insights in immune cell migration.

The surveillance function of immune cells requires both long-range patrolling throughout the body and local scanning of cells and molecular cues in tissues. For long-distance displacement, immune cells take advantage of the systemic blood and lymphatic circulation. Importantly, in the circulation, immune cells are also ensuring vessels integrity but how they detect anomalies and restore homeostasis remains poorly understood. In this Research Topic, Moreno-Cañadas et al. propose that intravascular crawling monocytes are switching from random migration to Levy-like motility upon detection of damaged endothelium. This motility mode alternates between fast & directed and slow & random migration phases, which enhances local patrolling. Interestingly, this type of motility has been previously reported as an optimal search strategy for T cells in infected brains (7). Adaptation of migratory behavior to local context in both health and disease is a common feature of immune cells. Tomas et al. review how the trafficking of monocytes and macrophages in arteries is altered during atherosclerosis as well as their contribution to return to homeostasis, highlighting the dual role of macrophages in both establishment and regression of the plaque. The role of immune cell circulation and adhesion to damaged vessels in disease onset is also illustrated in this topic by Morikis et al., who review the implication of neutrophils in vaso-occlusive crisis in sickle cell disease. Uncovering the molecular mechanisms linking immune cell migration and disease onset can help foster new therapeutic strategies, as suggested by these two reviews.

Exiting the systemic circulation requires immune cells to first detect exit signals, then adhere, roll and transmigrate from the circulation to the tissue interstitium (8). These processes are known to depend on integrin activation and conformational changes, but how integrin activation is fine-tuned remains to be fully understood (9, 10). Bromberger et al. investigate the role of the Rap1 and Riam pathways, converging to Talin1, on β2 integrin‐mediated rolling, adhesion and emigration of leukocytes. After transmigration, immune cells crawl under the endothelium before diving into the interstitial tissue. Arts et al. focus on this poorly-investigated step of immune cell migration and show that focal adhesions are avoided by neutrophils, hence determining their path underneath the epithelium.

Cues to exit the circulation and enter the interstitium are not only delivered by the vessels but also by the tissue calling for help. SenGupta et al. here review the secretome of cancer cells and its importance in triggering neutrophil invasion. Understanding how this secretome is generated and how neutrophils integrate the different cues to migrate could open new therapeutic options. Once in the tissue, the environment could drastically impact immune cell migration and function. This is particularly the case in tumors, where the stroma negatively modulates dendritic cell migration hence dampening T cell antitumoral activities, as reviewed by Gupta et al.

As evidenced in this topic, immune cells are facing highly distinct environments during their migratory journey throughout the organism. Yet a common denominator emerges, from hematopoiesis in primary lymphoid organs, to homing and local patrolling in secondary lymphoid organs and peripheral tissues. The extracellular cues of the tissue environment are finely integrated by immune cells through the actin cytoskeleton that controls their migration patterns. Kamnev et al. review how the study of immune actinopathies has contributed to our understanding of immune cell migration at the organism, tissue, cellular and molecular levels, highlighting the central role of the actin cytoskeleton and its regulators. The actin-related protein Arp2/3 complex is implicated in the coordination of migration and environment sensing in many different immune cell types (11–16). Investigating neutrophils from immunodeficiency patients with mutations in the Arp2/3 subunit ARPC1B, Kempers et al. here provide evidence for a differential role of ARPC1B in transmigration under flow shear stress and during interstitial human neutrophil migration. But actin is not the only important player, and the fine regulation of immune cell migration also lies in its interplay with the microtubule network. In this topic, Fazil et al. report the role of Glycogen synthase kinase 3β in T cell motility through interactions with the cytoskeletal regulator CRMP2.

Another important question addressed in this Research Topic is how very diverse extracellular cues are integrated by immune cells to turn on their cytoskeleton? Harcha et al. propose the pannexin channels as central sensors for both physical and chemical signals during inflammation. Having common receptors for distinct cues and converging signaling pathways could facilitate integration of information by immune cells.

Reviews and original articles in this Research Topic illustrate how advances in imaging microscopy over the last 20 years have shed light on the complex behavior of immune cells *in vivo* and contributed to our better understanding of molecular mechanisms underlying immune cell migration. Yet, a lot remains to be investigated and new models are a never-ending demand. First, primary immune cells present some limitations for mechanistic studies, as they may not survive well *in vitro*, or may not be amenable for genetic engineering. Uetz-von Allmen et al. propose a human neoplastic cell line CAL-1 as a good proxy for human dendritic cell signaling and migration. On the other hand, “just” observing cells is no longer sufficient to uncover molecular mechanisms underlying immune cell migration. The opto-genetic revolution offers new tools to fine-tune molecular activity that will be instrumental for deeper investigation. Amitrano et al. develop such a tool based on light stimulation to remotely control mitochondria activity and take advantage of it to reveal the role of mitochondrial ATP-production in CD8 T cell migration and function.

This Research Topic stresses the importance of better understanding immune cell migration in health and disease. A lot is known but a lot remains to be understood. Gaining mechanistic insights in the different physio-pathological contexts will open new avenues for innovative therapeutic strategies.

## Author Contributions

HM wrote the first draft of the editorial. JR and ED edited the manuscript. All authors contributed to the article and approved the submitted version

## Funding

HM is supported by the program “Investissements d’Avenir” launched by the French Government and Agence Nationale de la Recherche (ANR-20-CE15-0023 *InfEx*). ED is supported by the French Ligue Nationale contre le Cancer (Equipes labellisées). JR is supported by the Stiftung Experimentelle Biomedizin (Peter Hans Hofschneider Professorship), the LMU Institutional Strategy LMU-Excellent within the framework of the German Excellence Initiative, and the Deutsche Forschungsgemeinschaft (DFG; German Research Foundation; Collaborative Research Center SFB914, project A12; Priority Programme SPP2332, project 492014049).

## Conflict of Interest

The authors declare that the research was conducted in the absence of any commercial or financial relationships that could be construed as a potential conflict of interest.

## Publisher’s Note

All claims expressed in this article are solely those of the authors and do not necessarily represent those of their affiliated organizations, or those of the publisher, the editors and the reviewers. Any product that may be evaluated in this article, or claim that may be made by its manufacturer, is not guaranteed or endorsed by the publisher.
